# Mechanistic Insight into Etching Chemistry and HF-Assisted Etching of MgO-Al_2_O_3_-SiO_2_ Glass-Ceramic

**DOI:** 10.3390/ma11091631

**Published:** 2018-09-06

**Authors:** Yanxin Ji, Shun Yang, Zhulian Li, Junjie Duan, Meng Xu, Hong Jiang, Changjiu Li, Yongjun Chen

**Affiliations:** 1 State Key Laboratory of Marine Resource Utilization in South China Sea, Hainan University, Haikou 570228, China; jiyanxin14@gmail.com (Y.J.); yangshunchiyang@gmail.com (S.Y.); zhulianli123@sina.com (Z.L.); jungle_d@163.com (J.D.); xumeng691214@126.com (M.X.); jianghong@hainu.edu.cn (H.J.); chenyj99@163.com (Y.C.); 2 Special Glass Key Lab of Hainan Province, Haikou 570228, China; 3 Key Laboratory of Advanced Materials of Tropical Island Resources of Ministry of Education, Haikou 570228, China

**Keywords:** glass-ceramic, hydrofluoric acid, etching condition, etch rate, mechanism

## Abstract

The present study focuses on the etching conditions and mechanism of MgO-Al_2_O_3_-SiO_2_ glass-ceramic (MAS) in hydrofluoric acid (HF). The results show that the amorphous phase has 218 times higher etching rate than pure cordierite crystal at room temperature. In addition, the activation energies of cordierite and amorphous phases in the HF solution are 52.5 and 30.6 kJ/mol, respectively. The time (*t_ad_*) taken for complete dissolution of the amorphous phase depends on the HF concentration (*C_HF_*). Based on the etching experiments, a new model is established and refined to assess the *t_ad_* evolution. In addition, a highly crystalline cordierite phase, with the high specific surface area (59.4 m^2^·g^−1^) and mesoporous structure, has been obtained by HF etching. This paper presents novel insights into the etching chemistry and opens up avenues for further research in the area of cordierite-based catalytic ceramics.

## 1. Introduction

Cordierite ceramic is an excellent choice as a catalytic converter substrate for automobile exhaust purification due to its outstanding properties such as low thermal expansion coefficient, high chemical durability, and desirable refractories [1,2,3]. However, the relatively lower specific surface area of conventionally sintered cordierite ceramic results in a lower catalytic efficiency [4]. As a counter strategy, the cordierite ceramics are generally deposited by an active coating prior to the depositing of the catalytic layer [5,6]. In our previous research, a pine-like dendritic structure cordierite, as the main crystal phase, has been prepared based on MgO-Al_2_O_3_-SiO_2_ glass-ceramic (MAS), and the size of crystal gap in the dendritic cordierite is nanoscale [7]. He et al. enlarges the surface area of porous cordierite ceramic prepared by the compression molding method to 19.47 m^2^·g^−1^ by acid treatment [8].

With a well-known etching effect on silicate glasses [9,10,11,12,13,14], HF can be a feasible candidate to dissolve the amorphous phase with silicon-oxygen tetrahedron, the same as for the cordierite phase in MAS as well. Thus, in order to maximize the erosion degree of the amorphous phase and minimize the cordierite phase by HF as far as possible, it is necessary to study the etching conditions of MAS in HF so as to control the etching process, improve the microstructure of the crystallization phase, and to expand the application fields of glass-ceramics. However, few studies have been reported on either the etching conditions or mechanism of MAS in the HF solution. Lee et al. revealed that the microstructure and ration of the crystallinity of the cordierite phase could be controlled by changing the HF-etching conditions followed by a heating treatment process [15].

For precisely controlling the chemical etching process, the main factors affecting the etch rate and etch time are investigated. The origin of the etch rates gap between the two phases has also been discussed. Moreover, the relationship between the tad and *C_HF_* has been studied and a refined model has been established. We believe that it may be a good idea to prepare some glass ceramics with a higher specific surface area.

## 2. Materials and Methods

The 20MgO-20Al_2_O_3_-54SiO_2_-3K_2_O-1Fe_2_O_3_-2TiO_2_ (in molar %) glass was prepared from analytical purity (AR) chemical oxide and carbonate by melting at 1580 °C for 2 h in an alumina crucible in an electric furnace. The re-melted glass (10 cm × 10 cm × 1 cm) was cast on a metal block and placed in an annealing furnace, which was preheated to 650 °C. Then, the furnace was cooled to 500 °C (cooling rate 1 K/min), switched off, and cooled to room temperature. The glass samples were nucleated at 798 °C for 4 h, and crystallized at 945 °C for 4 h to achieve the MAS, respectively. During this process, the nucleations have been precipitated first when the nucleations are stable, the atoms in the melt migrate to the interface, making the crystals grow. Crystallized samples were grinded to powder with a particle size less than 75 μm and stored in the drying oven. For facilitating the comparison of etch rates between the amorphous phase and residual cordierite phase, the samples selected were the initial glass (made by melting) and pure cordierite crystal (HanYe Refractory, Shandong, China), respectively.

HF solutions with different concentrations are diluted by HF (40% *w*/*w*) with a purity grade of AR. All concentrations mentioned are in molar per liter (mol/L). Several batch experiments of *C_HF_* and time is designed to achieve the etch rate and *t_ad_* (as tabulated in Table 1). In this measurement, the etch time interval is 5 min. In each batch, 100 mg crystallized powder sample was added into 100 mL HF solution in a plastic tube to ensure the HF is in extreme excess so that the HF solution concentration changes are negligible. Substances in tubes are centrifuged for 5 min with a rotating speed of 3000 revolutions per minute. Achieved powder is dried in an oven at 80 °C.

The crystal structure of the samples were determined by X-ray diffraction (XRD, D8 Advance X, Bruker, Aachen, Germany). The XRD analysis was performed at a scanning speed of 2°/min, with a 0.01° step size, and Cu Kα radiations in the 2θ range of 5° to 70°. The microstructure of the samples were recorded by using scanning electron microscopy (SEM, MIRA3, TESCAN, Brno, Czech Republic). The specific surface area of the samples were measured by Brunauer-Emmett-Teller (BET, BK112T, Beijing JWGB Sci. Tech. Co., Ltd., Beijing, China). The mass loss (Δ*m*) was measured by an analytical balance, with an accuracy of ±0.1 mg. The relative content of the crystalline cordierite phase was obtained by dividing the mass of etched samples with the initial sample mass in a given time (*t*). The etch rate (*r*) can be extracted from the slope of the Δ*m*-time curve and depends on *C_HF_* [16].

(1) $$r=\frac{dm}{S\cdot dt}=k\cdot C_{HF}^{n}\;$$

where *k* (s^−1^) corresponds to the rate constant of the reaction, *n* represents the order of reaction and *S* refers to the surface area. As the value of the surface area is not constant in such measurements, it cannot be used to calculate exact etching. However, based on the initial BET surface area values, the surface area of the initial glass sample (1 m^2^/g) and the single phase cordierite (60 m^2^/g) has a value of 0.1 m^2^ and 6 m^2^, respectively, in the etching rate counts.

## 3. Results and Discussion

### 3.1. Phase and Microstructure Analysis

**Figure 1:**
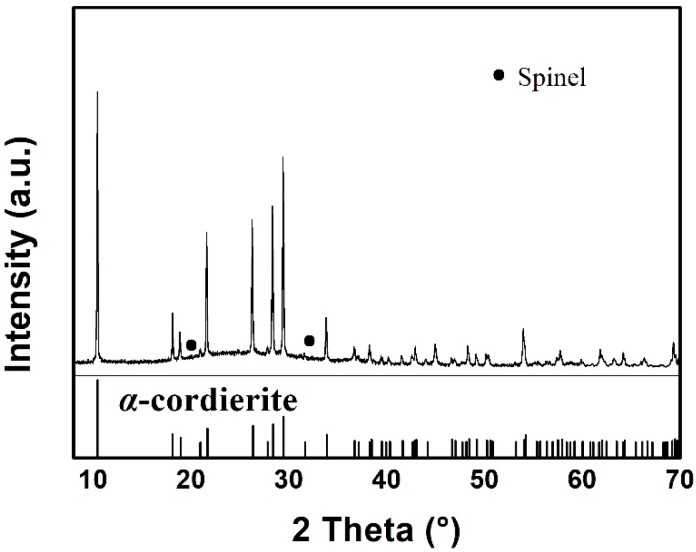
X-ray diffraction patterns of MAS.

**Figure 2:**
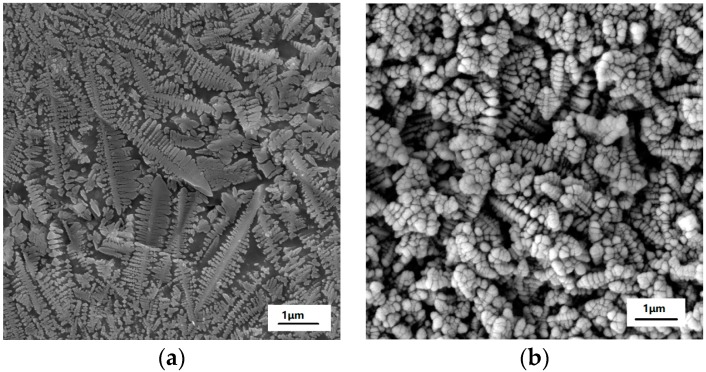
SEM images of MAS in etching conditions of (**a**) 5 mol/L HF for 10 s and (**b**) 20 mol/L HF for 30 min.

Figure 1 presents the XRD pattern of the as-prepared MAS, which exhibits the hexagonal α-cordierite (PDF#48-1600), which is the dominant phase with the space group of D_6h_^2^ = P6/mcc. The ratio of the amorphous versus crystalized phase can be approximated to 7:13. Additionally, low-intensity spinel phase peaks, at 2θ = 20° and 32°, can be discerned due to the presence of TiO_2_, which has been added as a nucleation agent in the raw material. The spinals always precipitate along with the main crystalline phase in MgO-Al_2_O_3_-SiO_2_ glass ceramics. Escobar J. et al. reported that the addition of TiO_2_ could inhibit the formation of the spinel-like species [17,18]. Therefore, a negligible amount of the spinel phase has been detected by XRD. Figure 2 shows the SEM images of MAS samples with different etching conditions. In the glass-ceramics, cordierite crystals are surrounded by an amorphous phase. Figure 2a presents the typical dendritic structure of MAS after etching for 10 s in 5 mol/L HF solution. Figure 2b indicates the over-etching of samples in 20 mol/L HF solution, which was carried out for 30 min. The SEM images indicate that optimal etching time should be adopted to avoid microstructural degradation.

### 3.2. Etching Process Analysis

**Figure 3:**
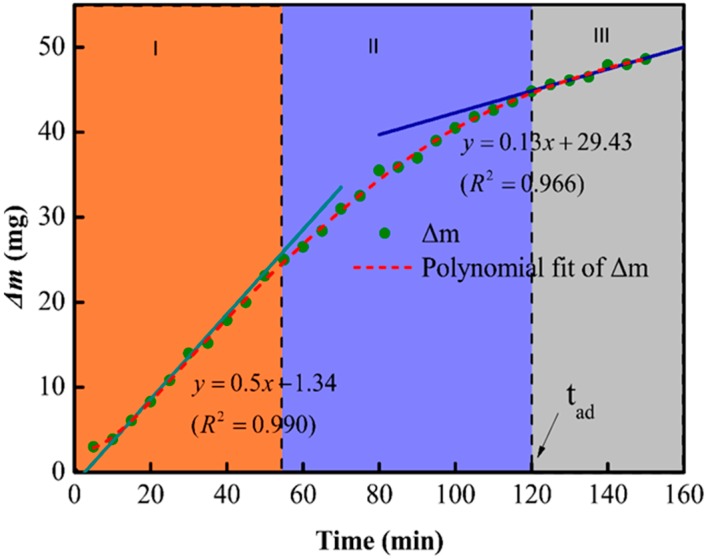
The relationship between change in the mass (delta-m) and time of MAS samples.

The etching mechanism was further investigated by establishing a relationship between etching time and the mass loss (Δ*m*) of MAS in 1 mol/L HF; the results are shown in Figure 3. The change in the slope of the curve indicates that the etching rate was not constant and decreased with time. In addition, the dissolution of glass-ceramic in HF can be divided into three regions: (1) The etching rate remained constant from 0 to 55 min, (2) the etching rate got reduced due to the dissolution of crystalline phases from 55 to 120 min, and (3) the etching rate attained a lower but constant value after 120 min due to the complete consumption of the amorphous phases. One should note that the gradual dissolution of the amorphous phase enhanced the exposure of the cordierites structure, which resulted in a lower etching rate in the second and third regions.

One should note that, despite the similar composition, the etching rate of crystalline (*r_c_*) and amorphous phases (*r_a_*) in HF solutions are different. The first and third regions of the mass-loss plot can be fitted with linear curves, which indicates the complex nature of glass ceramic reactions in HF solutions. According to Equation (1), *r_a_* and *r_c_* can be obtained by dividing the slope of fitted lines by the specific surface area. In this measurement, *r_a_* is 218 times faster than *r_c_* at room temperature.

**Figure 4:**
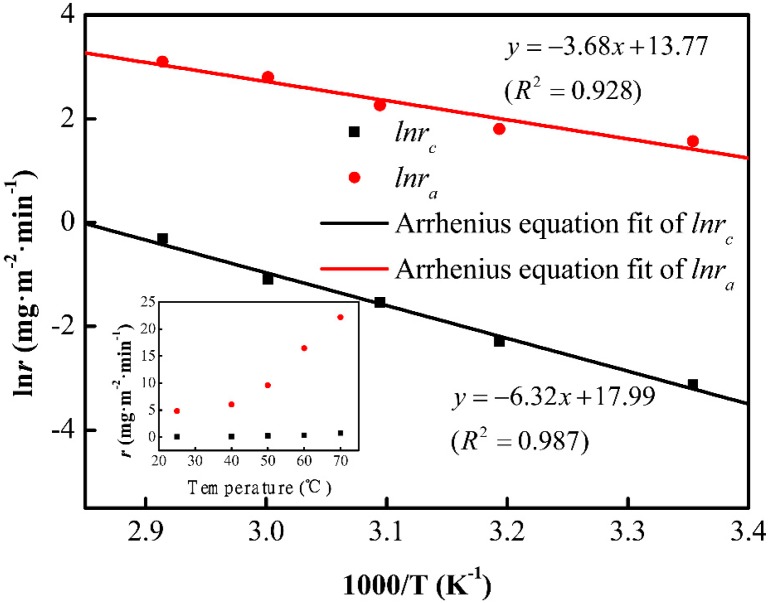
The etch rates of cordierite and amorphous phase fitted by the Arrhenius equation. Inset: The etch rates evolution depending on temperature.

To investigate the origin of divergence in etching rates, the etching rates of the cordierite phase and initial glass phase, at different temperatures, are shown in the inset of Figure 4. The etching rates have shown a direct relationship with temperature. The rate constant (*k*) can be expressed as a function of the reciprocal of temperature according to Arrhenius equation:

(2) $$k=A\cdot \exp(-\frac{Ea}{RT})\;$$

where *E_a_* refers to the activation energy, *R* = 8.314 J·mol^−1^·K^−1^ represents the molar gas constant, *T* corresponds to the thermodynamic temperature, and *A* refers to the pre-exponential factor. Equation (3) can be deduced from Equations (1) and (2):

(3) $$\ln r=-\frac{Ea}{RT}+\ln A+n\cdot \ln C_{HF}\;$$

where the total of ln*A* and *n*·ln*C_HF_* is a constant.

The chemical reaction rate is closely related to the activation energy of the reactants. Figure 4 presents the ln*r* vs. 1000/T curves. The activation energies of the cordierite and initial glass are 52.5 and 30.6 kJ/mol, respectively. It has been reported that the activation energy of vitreous SiO_2_ is about 30–32 kJ/mol [19]. Based on the activation energy, the reaction between the cordierite phase and HF is harder than the reaction between HF and the initial amorphous phase.

**Figure 5:**
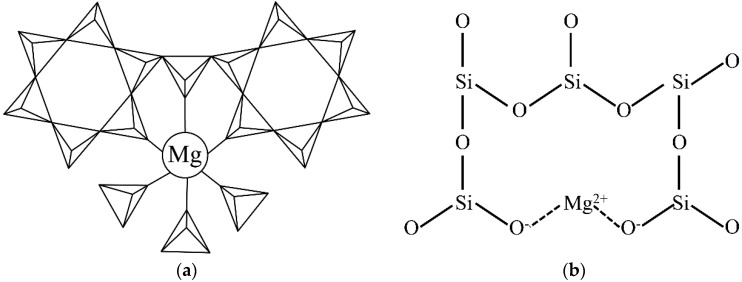
Two-dimensional diagram of different Mg^2+^ connection in (**a**) cordierite (triangles with lines inside represent [SiO_4_] and [AlO_4_] tetrahedrons) and (**b**) the amorphous phase (The Si–O bond perpendicular to the paper is not shown for watching convenience).

The cordierite crystal structure is mainly composed of [SiO_4_] tetrahedrons, [AlO_4_] tetrahedrons, and [MgO_6_] octahedrons, which are shown in Figure 5a. In the hexagonal α-cordierite, the [AlO_4_] occupies two random positions of the hexagonal rings and the remaining positions are occupied by [SiO_4_]. The hexagonal rings are connected by [AlO_4_] and [MgO_6_] to form a stable cordierite structure [20]. In the hexagonal α-cordierite, Mg^2+^ ions exist in the octahedral gaps and the coordination number is six. In the cordierite, the Mg–O bonds are covalent bonds [21,22]. However, the connection of Mg^2+^ ions is quite different in the amorphous phase (as shown in Figure 5b). In the amorphous phase, Mg^2+^ ions break up the SiO_2_ network and Si atoms are induced, which are bonded with less than four bridging oxygen atoms. The non-bridged oxygen atoms are terminated by Mg^2+^ ions and the silicon bonded to those oxygen atoms etched at a faster rate, similar to Si-F units. The two newly generated non-bridged oxygen atoms are linked by Mg^2+^ ions that resemble ionic bonds [23], which are weaker than the covalent bonds in the cordierite. Therefore, the etching rate of the amorphous phase is higher than the cordierite phase.

### 3.3. Factors Affecting t_ad_

It is crucial to control the reaction process and etching rate to reduce the erosion of the cordierite phase and to maximize the removal of the amorphous phase during HF treatment. Therefore, it is necessary to measure the degree of the amorphous phase removal. The complete removal of the amorphous phase is defined by the etching terminal point (*t_ad_*), which can be expressed as follow:

(4) $$t_{ad}=\frac{\Delta m}{S\cdot r}=\frac{\Delta m}{S\cdot k_{0}\cdot C_{HF}^{n_{0}}}\;$$

where *S* has an estimated average value of 0.05 m^2^.

**Figure 6:**
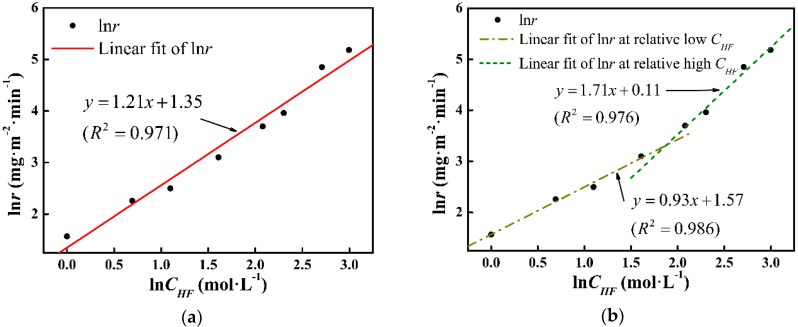
Arrhenius fitting of ln*r* in ln*C_HF_* domain at (**a**) whole range and (**b**) department ranges.

**Figure 7:**
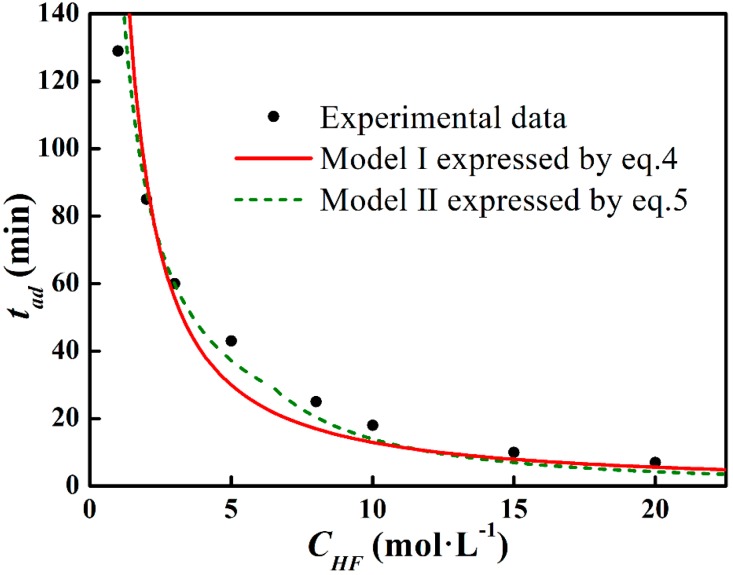
Data fit on Model I and II.

In Figure 6b, two fitting lines at different ln*C_HF_* ranges are drawn, and *t_ad_* can be expressed as:

(5) $$t_{ad}=\frac{\Delta m}{S\cdot k_{i}\cdot C_{HF}^{n_{i}}}(C_{HF}\leq 6.55,i=1;C_{HF}>6.55,i=2)\;$$

with *n*_1_ = 0.93, *k*_1_ (s^−1^) = 4.81, and *n*_2_ = 1.71 and *k*_2_ (s^−1^) = 1.12. Model II (expressed by Equation (5)) is also shown in Figure 7. The deviation has been minimized in Model II. To precisely define the etching terminal point, the tad function will be further corrected in a later study.

According to Equation (1), Figure 6a presents the Arrhenius curve of ln*r* vs. ln*C_HF_*. The slope of the fitting line corresponds to the reaction order and the intercept exhibits the logarithm of the rate constant, which are *n*_0_ = 1.21 and *k*_0_ = 3.86, respectively. Model I (expressed by Equation (4)) and the experimental *t_ad_* data are shown in Figure 7. It can be seen that the measured data fit Model I in low and high *C_HF_* regions, whereas a deviation is noticed in the middle *C_HF_* region, which corresponds to the variation of *n* value. 

### 3.4. Specific Surface Area Analysis

**Figure 8:**
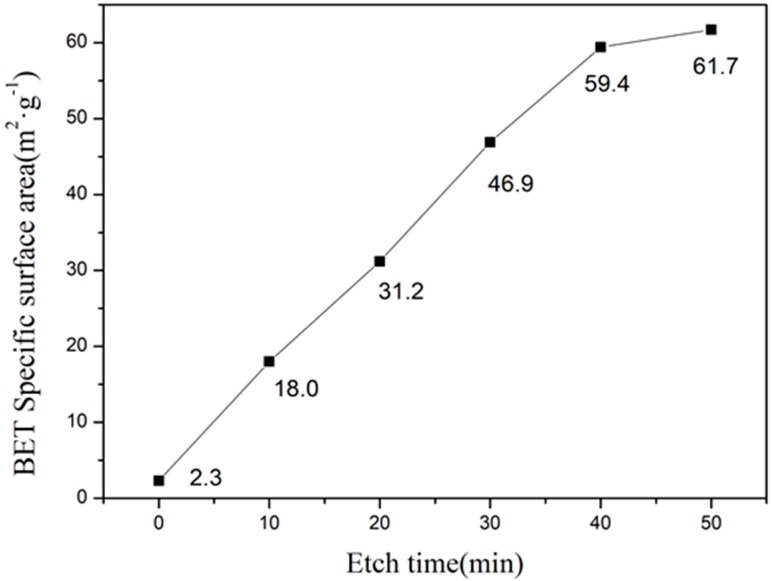
The relationship diagram of BET surface area and etching time.

Figure 8 shows the specific surface area of as-prepared and etched samples. We have observed a linear relationship between the etching time and specific surface area. However, after 40 min of etching, the increase in specific surface area was slower due to the complete dissolution of the glass phase. In addition, etching for 50 min resulted in higher specific surface area due to over-etching, which completely dissolved the cordierite phase. Furthermore, the specific surface area of 59.4 m^2^·g^−1^ has been obtained after 40 min of etching, which is 10 times higher than the as-prepared sample.

**Figure 9:**
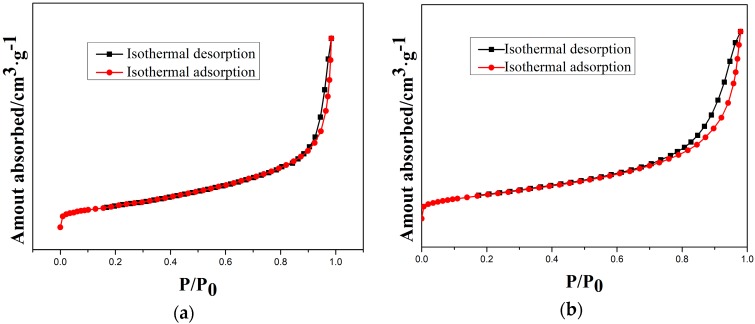

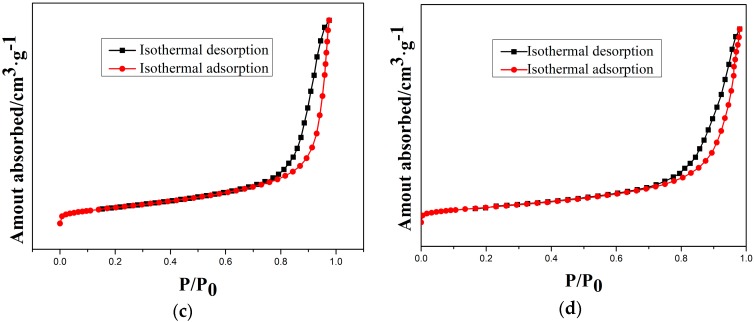
N_2_ isothermal absorption and desorption curves of samples at different etching times: (**a**) 10 min; (**b**) 20 min; (**c**) 30 min; and (**d**) 40 min.

Moreover, the type-IV isotherms (Figure 9) indicate the mesoporous structure of as-prepared and etched samples. Therefore, a cordierite phase with the high specific surface area and mesoporous structure can be obtained by optimal etching conditions.

## 4. Conclusions

Herein, we have investigated the dissolution of cordierite glass-ceramic in HF solution and obtained a highly crystalline cordierite phase with the high specific surface area and mesoporous structure. We have observed significant differences between etching rates of cordierite and the amorphous phase. In 1 mol/L HF solution, *r_a_* was ~218 times higher than *r_c_*, which has been explained by using the activation energy values of cordierite (52.5 kJ/mol) and the amorphous (30.6 kJ/mol) phases. In the amorphous phase, Mg^2+^ ions broke the SiO_2_ network and resulted in a loose structure.

Furthermore, a new model for the *t_ad_* evolution has been established and refined by the fitted reaction orders as:

(6) $$t_{ad}=\frac{\Delta m}{S\cdot k_{i}\cdot C_{HF}^{n_{i}}}(C_{HF}\leq 6.55,i=1;C_{HF}>6.55,i=2)\;$$

Interestingly, the specific surface of the cordierite area has been improved to 59.4 m^2^·g^−1^ by using optimal HF etching conditions.

## Figures and Tables

**Table 1:** Etching conditions of MAS in HF solution

*C_HF_* (mol/L)	Etch Time (min)
1	5–200
2	5–200
3	5–200
5	5–200
8	5–200
10	5–200
15	5–200
20	5–200
